# The SREBP (Sterol Regulatory Element-Binding Protein) pathway: a regulatory bridge between carotenogenesis and sterol biosynthesis in the carotenogenic yeast *Xanthophyllomyces dendrorhous*

**DOI:** 10.1186/s40659-021-00359-x

**Published:** 2021-10-26

**Authors:** Melissa Gómez, Marcelo Baeza, Víctor Cifuentes, Jennifer Alcaíno

**Affiliations:** 1 Departamento de Ciencias Ecológicas, Facultad de Ciencias, Universidad de Chile, Las Palmeras 3425, Santiago, Chile; 2 Centro de Biotecnología, Facultad de Ciencias, Universidad de Chile, Las Palmeras 3425, Santiago, Chile

**Keywords:** SREBP/Sre1, Sterols, Astaxanthin, Carotenoids, Fungi

## Abstract

*Xanthophyllomyces dendrorhous* is a basidiomycete yeast that naturally produces the red–orange carotenoid astaxanthin, which has remarkable antioxidant properties. The biosynthesis of carotenoids and sterols share some common elements that have been studied in *X. dendrorhous*. For example, their synthesis requires metabolites derived from the mevalonate pathway and in both specific pathways, cytochrome P450 enzymes are involved that share a single cytochrome P450 reductase, CrtR, which is essential for astaxanthin biosynthesis, but is replaceable for ergosterol biosynthesis. Research on the regulation of carotenoid biosynthesis is still limited in *X. dendrorhous*; however, it is known that the Sterol Regulatory Element-Binding Protein (SREBP) pathway, which is a conserved regulatory pathway involved in the control of lipid metabolism, also regulates carotenoid production in *X. dendrorhous.* This review addresses the similarities and differences that have been observed between mammal and fungal SREBP pathways and what it is known about this pathway regarding the regulation of the production of carotenoids and sterols in *X. dendrorhous*.

## Background

**Fig. 1:**
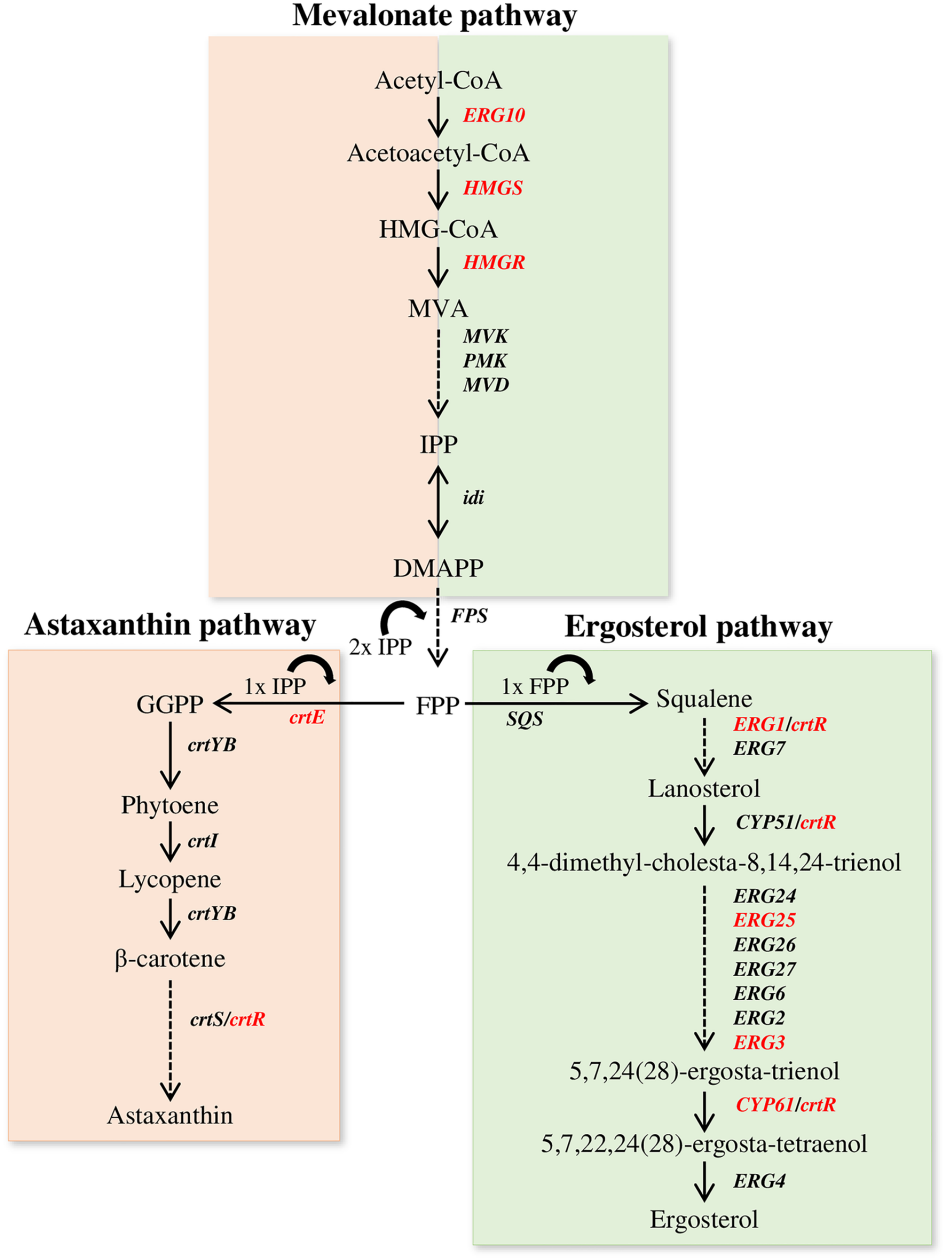
Biosynthesis of astaxanthin and ergosterol in *X. dendrorhous*. The production of carotenoids and sterols requires isopentenyl pyrophosphate (IPP) from the mevalonate pathway [9]. Carotenoid biosynthesis begins with the production of geranylgeranyl pyrophosphate (GGPP) from farnesyl pyrophosphate (FPP) and IPP by GGPP synthase encoded by *crtE* [66, 68]. Then, the bi-functional enzyme phytoene-β-carotene synthase encoded by *crtYB* condenses two GGPP molecules producing phytoene [69]. Subsequently, phytoene undergoes four desaturation reactions carried out by the phytoene desaturase enzyme encoded by *crtI*, producing lycopene [70]. The cyclization of both ends of lycopene by the lycopene cyclase activity of the phytoene-β-carotene synthase produces β-carotene [69], which is then transformed into astaxanthin via intermediate xanthophylls. To date, *X. dendrorhous* is the only known organism that produces astaxanthin from β-carotene through a cytochrome P450 system [71], which is composed of the cytochrome P450 enzyme astaxanthin synthase (CrtS, encoded by *crtS*) [72, 73] and a cytochrome P450 reductase (CPR) (named CrtR in *X. dendrorhous*, encoded by *crtR*) [64]. CrtS catalyzes the hydroxylation and ketolation of carbons at positions 3 and 4, respectively, of each end ring of β-carotene to finally produce astaxanthin [72], while CrtR assists CrtS in these reactions [64]. In ergosterol biosynthesis, two cytochrome P450 enzymes are involved, which are encoded by *CYP51* (lanosterol 14α-demethylase) [74] and *CYP61* (C-22 sterol desaturase) [49]. Arrows: catalytic steps with the corresponding enzyme encoding gene. Genes in red: Direct Sre1 targets identified by ChIP-exo [17]. CrtR was included as a redox partner of the cytochrome P450 monooxygenases Cyp51, Cyp61 and CrtS, and squalene epoxidase (*ERG1*). 3-hydroxy-3-methylglutaryl-CoA (HMG-CoA), mevalonate (MVA), dimethylallyl-pyrophosphate (DMAPP). Other genes: *ERG10* (acetyl-CoA C-acetyltransferase), *HMGS* (HMG-CoA synthase), *HMGR* (HMG-CoA reductase), *MVK* (mevalonate kinase), *PMK* (phosphomevalonate kinase), *MVD* (mevalonate diphosphate decarboxylase), *idi* (IPP isomerase), *FPS* (FPP synthase). *ERG* represent to enzyme-encoding genes involved in ergosterol biosynthesis identified in *Saccharomyces cerevisiae*. Figure adapted from [17]

The use of microbial platforms as natural carotenoid producers has gained interest as an alternative to the production of carotenoids by chemical synthesis due to health concern of synthetic carotenoids [1]. Even the cost associated with the production of carotenoids using microorganisms could be reduced using agro-industrial wastes as low-cost substrates, which also have an ecological aspect [2, 3]. The basidiomycetous yeast *Xanthophyllomyces dendrorhous* is a natural source of astaxanthin. This red–orange carotenoid has remarkable antioxidant activity [4] and is gaining increasing attention as evidence indicates that it is an effective molecule preventing oxidative stress-mediated and age-related diseases [5]. Astaxanthin is also used in aquaculture as a food additive, principally because it improves muscle pigmentation of aquatic species, and subsequently product quality and price [6]. There are several reviews related to astaxanthin biosynthesis in *X. dendrorhous* [7–10] that make this yeast one of the best candidates for commercial production of astaxanthin; however, this process has not been economically efficient to date [11]. On the other hand, research on the mechanisms regulating the carotenoid production, which could be used as a tool to improve the production of carotenoids in *X. dendrorhous*, is still limited. It is known that astaxanthin production is regulated by environmental and nutritional conditions. For example, reactive oxygen species induce carotenoid production, which is probably related to the antioxidant protective role of carotenoids [12–14]. At the nutritional level, the production of carotenoids in *X. dendrorhous* is regulated by glucose‐dependent repression, depending on the DNA‐binding regulator Mig1 and the co‐repressor complex Cyc8-Tup1, which repress the expression of several genes including genes involved in carotenogenesis [15, 16]. Recently, it has been demonstrated that carotenogenesis in *X. dendrorhous* is regulated by the SREBP (Sterol Regulatory Element-Binding) pathway [17]*,* which is a conserved pathway in several organisms involved in lipid homeostasis that is activated depending on sterol demand [18]. In *X. dendrorhous*, this pathway regulates several genes of the mevalonate (MVA) pathway and from ergosterol biosynthesis; interestingly, it also regulates two carotenogenic genes (Fig. 1) [17]. Therefore, considering the aforementioned findings, the goal of this review is to gather and discuss the latest reports related to the SREBP pathway in fungi, focusing on the common aspects found in the regulation of the biosynthesis of sterols and carotenoids in *X. dendrorhous*.

## The SREBP pathway in mammals and in fungi

**Fig. 2:**
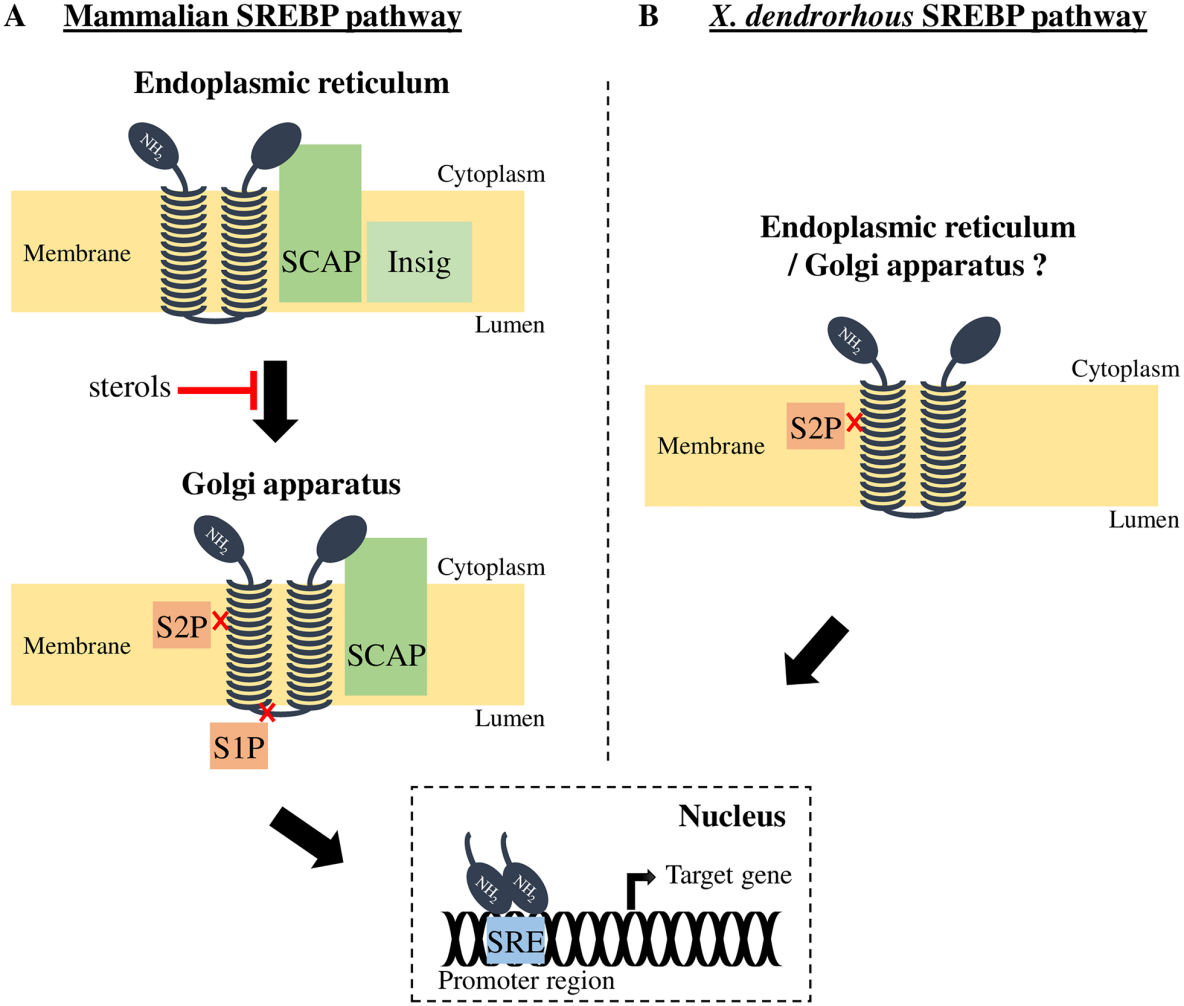
The SREBP pathway in **A** mammals and **B** *X. dendrorhous*. The SREBP pathway is involved in the regulation of lipid homeostasis and metabolism, and is regulated by several independent mechanisms including the use of subcellular compartmentalization to ensure its activation, when required. In sterol-replete mammalian cells, the Insig proteins (isoforms: Insig-1 and Insig-2) bind cholesterol-loaded protein SCAP to retain the SCAP-SREBP complex in the endoplasmic reticulum membrane. On the other hand, when cells are depleted of sterols, the SREBP-SCAP complex is transported to the Golgi apparatus where SREBP is sequentially processed by proteases S1P and S2P. As a result, the N-terminal domain (NH_2_) of SREBP, which contains the basic helix-loop-helix leucine zipper (bHLH-LZ) domain, is released, migrates to the nucleus, and binds as a dimer to the sterol regulatory elements (SREs) in the promoter region of target genes activating their transcription. In *X. dendrorhous*, the SREBP homolog is Sre1, which is processed by a S2P homolog, named Stp1. Figure adapted from [17]

The SREBP pathway is involved in the regulation of lipid homeostasis and metabolism, and our knowledge originates primarily from studies in mammalian cells. Therefore, it is necessary to highlight the key elements of the mammalian SREBP pathway (Fig. 2A), before establishing the differences found in fungi, specifically in *X. dendrorhous*. The central components of the SREBP pathway are the SREBP proteins, which are a family of basic-helix-loop-helix leucine zipper (bHLH-LZ) transcription factors that are initially synthesized as inactive precursors that are anchored to the Endoplasmic Reticulum (ER) membrane [19]. In mammals, three isoforms of these transcription factors have been described: SREBP-1a, SREBP-1c [20, 21] and SREBP-2 [22]. The first two are encoded by a single gene and differ due to the use of alternative transcription start sites. In all three cases, the precursor form is bound to the ER membrane in a harpin fashion with the NH_2_-terminal (the transcription factor domain, bHLH-LZ) and COOH-terminal (the regulatory domain) domains facing the cytoplasm. These terminal domains are separated by a short hydrophilic sequence (the lumenal loop) which projects into the lumen of the ER [23]. In the ER membrane, SREBP may form a complex with the SREBP cleavage-activating protein (SCAP), which itself harbors a sterol sensing domain [24]. Specifically, this interaction occurs between the WD repeat domain of the SCAP C-terminal domain and the SREBP C-terminal regulatory domain [25]. When sterol levels are sufficient, the Insig (Insulin induced gene) proteins bind to cholesterol-loaded SCAP protein to retain the SCAP-SREBP complex in the ER [26, 27]. On the other hand, when sterol levels decrease, the SCAP-SREBP complex clusters into COPII-coated vesicles that bud from the ER [28], which are then transported to the Golgi apparatus where SREBP is sequentially processed by proteolytic cleavage for activation in two steps. First, site-1 protease (S1P, a subtilisin-related serine protease) cuts SREBP in its ER lumenal loop [29] and next, site-2 protease (S2P, a metallopeptidase) hydrolyzes SREBP within the first transmembrane segment [30]. As a result, the N-terminal bHLH-LZ domain of SREBP is released and migrates to the nucleus where it activates the transcription of target genes by binding to Sterol Regulatory Elements (SREs) at their promoter region [21].

### Components of the SREBP pathway in ascomycete and basidiomycete fungi

SREBP-like protein encoding genes have been identified in several fungal genomes, and some have proven to be functional (Table 1). The SREBP pathway in fungi is also involved in lipid homeostasis and metabolism, and in some cases, it has been demonstrated that it is activated under low oxygen conditions regulating the hypoxic response. Most studies of fungal SREBP pathways have been carried out in the ascomycetes *Schizosaccharomyces pombe* [31] and species of the genus *Aspergillus* such as *Aspergillus fumigatus* [32], while studies in basidiomycetes have focused on *Cryptococcus neoformans* [33, 34]*.* In these fungi, the SREBP pathway is also involved in resistance to azole compounds, which are antifungal drugs that negatively affect ergosterol biosynthesis [35], and in pathogenesis in *A*. *fumigatus* and *C. neoformans* [32, 34]. Novel functions of the SREBP pathway in fungi include the maintenance of cell polarity in *A. fumigatus* [32], and the existence of a regulatory link in protein secretion under lignocellulolytic conditions in *Neurospora crassa* and *Trichoderma reesei* [36, 37]. Differences in the components of the SREBP pathway, including the proteolytic activation mechanism of the transcription factor SREBP, between the mammalian and fungal SREBP pathways have been detected, as well as dissimilarities between ascomycetes and basidiomycetes, and even differences within each division (Table 1).

**Table 1:** Components of SREBP pathways described in some fungi

Fungi division	Fungal species	SREBP pathway components						
		SREBP		SCAP	Insig proteins*	SREBP proteolytic activation		
						S1P	S2P	Other components involved in the activation of SREBP**
			DUF2014 domain					
Ascomycota	*S. pombe*	Sre1, Sre2 [31]	Present in Sre1 and missing in Sre2 [31]	Scp1 [31]	Ins1[31]	–	–	Dsc1 to Dsc4 [39]; Dsc5, Cdc48 [38]; Rbd2 [40]; Ypf1 [42]
	*A. fumigatus*	SrbA [32]; SrbB [45]	Present in SrbA [32], and missing in SrbB	–	InsA [75]	–	–	DscA to DscD [43]; RbdB [44]/RbdA [76]; homolog of *A. nidulans* SppA (ID AspGD database: Afu6g02150)
	*A. nidulans*	SrbA [42]; homolog of *A. fumigatus* SrbB (ID AspGD database: An7170)	Present in SrbA [42], and missing in homolog of *A. fumigatus* SrbB (GeneBank ID: CBF78935.1)	–	–	–	–	SppA, DscA to DscE [42]. Homolog of *A. fumigatus* RbdB/RbdA (GenBank ID: CBF75549.1)
	*N. crassa*	SAH-2/SRE-1 [37, 77]; SRE-2 [36]	Present in SAH-2 and missing in SRE-2 [36]	SCP-1 [36]	–	–	–	Dsc-1/TUL-1, Dsc-2 to Dsc-6, RBD-2, Spp-1 [36, 37]
Basidiomycota	*C. neoformans*	Sre1 [33]	Missing in Sre1 [51]	Scp1 [33]	–	–	Stp1 [34, 46]	–
	*X. dendrorhous*	Sre1 [48]	Missing in Sre1 [48]	–	–	–	Stp1 [47]	–

AspGD = The Aspergillus Genome Database. DUF2014 domain = domain of unknown function that is present at the C-terminal of some SREBP homologs (Pfam entry: PF09427)

*Insig proteins: *S. pombe* Ins1 is not essential for retention of the SCAP homolog in the ER [31]. The gene *insA* of *A. fumigatus* encodes a putative Insig protein (ID AspGD database: Afu4g07680) and *A. nidulans* has a hypothetical protein homolog of InsA (ID AspGD database: AN4465). No homologs were detected in *N. crassa* [36], *C. neoformans* [78], and *X. dendrorhous* [48]

**Other components involved in the activation of SREBP: SppA has conserved biological functions in *A. nidulans* and *A. fumigatus* [42]. *S. pombe* Ypf1 [42] and *N. crassa* Spp-1 [36], homologs to SppA are not involved in the response to hypoxia conditions

### Ascomycetes (*S. pombe*, species of the genus* Aspergillus *and *N. crassa*)

#### Schizosaccharomyces pombe

In the fission yeast *S. pombe,* homologs of SREBP, SCAP and Insig proteins have been identified (Sre1, Scp1 and Ins1, respectively) [31]. Interestingly, *S. pombe* has a second SREBP homolog (Sre2), whose role is still unknown [31]. The proteolytic activation of *S. pombe* Sre1 is mediated by a different mechanism to the one described in mammalian cells, as it lacks S1P and S2P homologs. Sre1 activation depends on the Golgi Dsc (defective for SREBP cleavage) E3 ligase complex (proteins Dsc1 to Dsc5; Dsc1 has E3 ubiquitin ligase activity) [38, 39] and rhomboid protein 2 (Rbd2) [40], that binds the AAA-ATPase Cdc48, which probably recognizes ubiquitinylated Sre1 and recruits it for Rbd2 cleavage. However, a second cut in Sre1 by another protease is not ruled out [41].

#### Species of the genus *Aspergillus*

In *Aspergillus,* the proteolytic processing of the SREBP homolog (SrbA) [32, 42] appears to be like that of *S. pombe* since *A. fumigatus* and *A. nidulans* also lack S1P and S2P homologs. In *A. fumigatus*, the homologs of the Golgi Dsc E3 ligase complex (DscA to DscD) [43], and rhomboid protease (RbdB) [44] are involved in SrbA processing. On the other hand, a second SREBP homolog identified in *A. fumigatus* (SrbB), is probably independent of proteolytic cleavage as no transmembrane domains are predicted in its structure [45]. Interestingly, *A. nidulans* SrbA is sequentially processed by Dsc-linked proteolysis followed by an aspartyl protease (SppA) [42]. Unlike the fission yeast, no homologs of SCAP have been detected in *Aspergillus*. Interestingly, a SppA homolog (Ypf1) was also detected in *S. pombe*, but it is not involved in the response to hypoxia conditions [42].

#### Neurospora crassa

Like *S. pombe* and *A. fumigatus*, *N. crassa* has homologs of SREBP (SAH-2 and SRE-2), SCAP (SCP-1), SppA (Spp-1), Rbd2 (RBD-2) and of components of the Golgi Dsc E3 ligase complex (Dsc-1 to Dsc-6) [36]. In this ascomycete, proteins SRE-2 and Spp-1 are not related to hypoxia adaptation and are therefore not required for the function of the SREBP pathway. Based on a model, the activation of SAH-2 probably involves homologs of the Golgi Dsc E3 ligase complex and rhomboid protease Rbd2 [36].

### Basidiomycetes (*C. neoformans *and *X. dendrorhous*)

#### *Cryptococcusneoformans*

In the case of the basidiomycete *C. neoformans*, unlike *S. pombe* and *Aspergillus*, it was shown that the SREBP homolog (Sre1) [33] is processed by a homolog of mammalian S2P (Stp1) [34, 46]. Interestingly, like *S. pombe* and *N. crassa*, this basidiomycete has a SCAP homolog, which was shown to be involved in the SREBP pathway [33].

#### Xanthophyllomyces dendrorhous

In the basidiomycete *X. dendrorhous,* SREBP and S2P homologs were recently described (Sre1 and Stp1, respectively), which were proven to be involved in the SREBP pathway in this yeast [47, 48] (Fig. 2B).

## The SREBP pathway in *X. dendrorhous*

*Xanthophyllomyces* *dendrorhous* produces the carotenoid astaxanthin and a carotenoid overproduction phenotype was observed in an ergosterol biosynthesis mutant, which was one of the first evidence that suggested that carotenoid production in this yeast could be regulated, at least in part, by the SREBP pathway. Cyp61 is a cytochrome P450 enzyme that catalyzes the second last step of ergosterol biosynthesis (Fig. 1), and carotenoid content increased approximately twofold compared to those of the wild-type strain when this gene was interrupted [49]. Recent works revealed that this phenotype depends on the SREBP pathway as it was demonstrated that the *X. dendrorhous* SREBP homolog was activated in the *cyp61*^−^ mutant and mutations that avoided Sre1 activation in this strain, brought carotenoid levels back to wild levels [47, 48]. This section summarizes our current knowledge of the SREBP pathway in *X. dendrorhous*.

### *Xanthophyllomyces dendrorhous* Sre1 is conserved and is probably activated independent of SCAP

*Xanthophyllomyces dendrorhous* Sre1 has the characteristic bHLH-LZ DNA binding domain with a tyrosine (Y364), which distinguishes SREBPs from other bHLH transcription factors [50]. It also lacks a DUF2014 domain [48], which is a domain of unknown function that is present at the C-terminal of some SREBP homologs and may be important for interaction between SREBP and SCAP, and therefore, for the ER membrane retention of SREBP [51]. However, a potential SCAP encoding gene is not distinguished in the *X. dendrorhous* genome, suggesting that this yeast lacks a SCAP homolog. As in *X. dendrorhous*, *C. neoformans* Sre1 also lacks the DUF2014 domain, but this basidiomycete does have a SCAP homolog (Scp1) [33]. *A. fumigatus* SrbA harbors the DUF2014 domain, but this ascomycete lacks a SCAP homolog [42]. Thus, it is possible that *X. dendrorhous* has a SCAP-independent Sre1 activation mechanism that could also be conserved in other fungi lacking SCAP, like the pathogen *A. fumigatus.* In mammals, the SCAP and the Insig proteins are important components of the SREBP pathway, as they bind cholesterol and cholesterol hydroxylated derivatives (such as 25-hydroxycholesterol), respectively [52, 53], and in this way they regulate SREBP retention at the ER membrane. Like *C. neoformans* and *Aspergillus*, *X. dendrorhous* lacks an Insig homolog, and, even though an Insig homolog was identified in *S. pombe*, it is not involved in the SREBP pathway of this yeast [31]. This evidence suggests that the SREBP pathways in fungi are Insig-independent and in some fungal species, this pathway may also be SCAP-independent, as in the case of *X. dendrorhous*.

### Sre1 is cleaved by Stp1, but the “sterol level sensor” and the “sterol-signal” that induce Sre1 activation by proteolytic cleavage in *X. dendrorhous* are still unknown

*Xanthophyllomyces dendrorhous* lacks a S1P protease homolog involved in Sre1 cleavage, but it is known that a S2P protease homolog (Stp1) is involved in this process [47]. A similar Sre1 activation mechanism was described in *C. neoformans*, as although it also lacks a S1P homolog, it does possess a S2P homolog involved in Sre1 activation [46]. However, the SCAP homolog of *C. neoformans* is required for Sre1 activation in response to lower sterol levels under low oxygen conditions [33]. In *S. pombe*, the Sre1-Scp1 complex senses ergosterol, and Sre1 transport and activation depends on the ergosterol concentration in the ER [54]. In *X. dendrorhous,* the sterol signal that favors Sre1 activation is still unknown. However, in mutants that do not produce ergosterol and overproduce carotenoids (*cyp61*^−^ mutants), Sre1 is mainly in its activated form (Sre1N, N-terminal bHLH-LZ domain of Sre1) under standard laboratory conditions, correlating with the higher transcript levels of genes regulated by Sre1 that may be responsible in part for the carotenoid overproduction phenotype [47]. However, other ergosterol biosynthesis mutants (mutants of genes *ERG3* and *ERG4*) that also do not produce ergosterol, do not overproduce carotenoids, and transcript levels of genes regulated by Sre1 are the same as in the wild-type strain [55]. These observations suggest that it is not the absence of ergosterol itself that triggers Sre1 activation in *X. dendrorhous*, but rather Sre1 activation depends on other alterations in sterol composition.

### Additional regulation mechanisms on SREBPs

Besides proteolytic activation, SREBPs are regulated by covalent modifications and by interaction with other proteins. This has been studied more in mammals than in fungi. In mammals, regulation of SREBP by phosphorylation-dependent degradation has been reported [56, 57]. For example, Glycogen Synthase Kinase—3 (GSK-3) has been implicated in the phosphorylation of the precursor form of SREBP-1c in a specific serine [58]. Thus, phosphorylated SREBP-1c has lower affinity for SCAP, and free SREBP-1c is targeted for degradation [58]. In *S. pombe*, the active Sre1 transcription factor (Sre1N) is regulated by a casein kinase 1 family member (Hhp2) that promotes Sre1N proteasomal degradation through phosphorylation of Sre1N at specific residues [59]. In the *X. dendrorhous* genome, genes encoding putative kinase homologs to *S. pombe* Hhp2 (CDZ96742.1) and to mammalian GSK-3 (CDZ96841.1) were identified, and several serine and threonine residues of Sre1N are predicted to be potential phosphorylation sites. However, studies are needed to determine if indeed phosphorylation by these potential kinases or by others has a role in the regulation of Sre1 in *X. dendrorhous*. Additionally, in *S. pombe* the protein Ofd1 (2-oxo-glutarate Fe(II) dioxygenase) accelerates Sre1N proteasomal degradation and inhibits Sre1N DNA binding under normoxic conditions [60]. *X. dendrorhous* has a potential Ofd1 homolog (CED84823.1), but it may not be involved in the *X. dendrorhous* SREBP pathway as the phenotype of strains that only produce the active Sre1 transcription factor was not modified with the *OFD1* mutation [61].

### Role of the SREBP pathway in *X. dendrorhous*

As a SREBP homolog, *X. dendrorhous* Sre1 is involved in lipid homeostasis and in resistance against antifungal drugs [47, 48], which is observed in *A. fumigatus* by the direct regulation by Sre1 of a *cdr1B* homolog gene [17] that encodes an ABC transporter that contributes to the resistance to azoles [62]. Interestingly, *A. fumigatus cdr1B* is regulated by AtrR*,* which is a transcription factor that also regulates the synthesis of ergosterol and shares target genes with SrbA [63]*.* At this point, it would be interesting to explore the existence and role of possible counterpart of AtrR in *X. dendrorhous*. Importantly, the SREBP pathway plays a role in carotenogenesis through the regulation of carotenogenic genes *crtE* and *crtR* (Fig. 1) [17]. In addition, small ChIP-exo peaks were observed in the promoter region of the astaxanthin synthase encoding gene (*crtS* gene), although no SRE elements were detected. However, when comparing transcript levels among strains having an activated SREBP pathway and *sre1*^−^ mutants by RNA-seq analysis, the transcriptional profile of *crtS* was similar to that of *crtR* (higher transcript levels in strains having an active SREBP pathway and lower levels in *sre1*^−^ mutants), suggesting that *crtS* might be regulated by Sre1 at some level [17]. CrtR is the only cytochrome P450s reductase in this yeast, and it participates in both: carotenoid (assisting the cytochrome P450 enzyme astaxanthin synthase, CrtS) and ergosterol (assisting the cytochrome P450 enzymes Cyp51 and Cyp61, and probably squalene epoxidase) biosynthesis (Fig. 1). However, CrtR is only essential for astaxanthin biosynthesis [64] as the *crtR*^*−*^ mutants of *X. dendrorhous* do not produce astaxanthin and accumulate β-carotene, but, although in a lower proportion when compared to the wild-type strain, they do produce ergosterol. This last observation supported that there is an alternative electron donor to the cytochrome P450 reductase enzyme (probably the NADH-dependent cytochrome b5 reductase and cytochrome b5 via) that in *X. dendrorhous* may assist the production of ergosterol, but not that of astaxanthin [65]. The regulation of *crtR* by Sre1 is proof of links between carotenoid and sterol production in *X. dendrorhous.*

On the other hand, carotenogenesis depends on metabolites from the MVA pathway. It is important to note that in non-carotenogenic organisms, the MVA pathway is usually considered as the first steps of the sterol biosynthesis pathway. Therefore, these genes are generally considered as genes of sterol biosynthesis. However, products of the MVA pathway are also precursors of other metabolites than sterols; for example, carotenoids in non-photosynthetic organisms as in *X. dendrorhous.* In *X. dendrorhous*, Sre1 directly regulates the transcription of three genes of the MVA pathway (Fig. 1). Overexpression of genes of the MVA pathway and of gene *crtE* in *X. dendrorhous*, favors carotenoid production [66, 67]; therefore, the regulation of genes of the MVA pathway and is another evidence of regulatory bridging between the production of carotenoids and sterols in this yeast.

## Conclusions

The SREBP pathway in fungi is still not fully understood and current knowledge is based on studies on few fungal models. In ascomycetes, the proteolytic activation of the SREBP homolog depends on several components of the SREBP pathway, which include proteins that anchor SREBP to the ER membrane and mechanisms that include at least one protease involved in the processing of SREBP. In *X. dendrorhous*, like the basidiomycete *C. neoformans*, Sre1 cleavage depends on the mammalian S2P homolog, the Stp1 protease. However, after the first hydrolysis performed by Stp1, the possibility of a second cleavage of Sre1 by another protease is not ruled out*.* On the other hand, unlike *C. neoformans, X. dendrorhous* lacks a SCAP homolog, which is the sterol sensor protein that retains SREBP in the ER membrane. Thus, Sre1 processing in *X. dendrorhous* is probably SCAP-independent and depends on sterol composition changes that are still unknown. This mechanism is different from what is observed in mammals and in some fungi, where the transcription factor is activated in response to sterol depletion in a SCAP-dependent manner. These differences raise important questions in *X. dendrorhous* about how this yeast monitors sterol levels and regulates the stability and proteolytic activation of Sre1. On the other hand, it is likely that Sre1 in *X. dendrorhous* is also regulated by post-translational modifications, as reported in the yeast *S. pombe*; however, this has not yet been explored in the carotenogenic yeast. Another difference between members of the ascomycota and basidiomycota divisions is the presence of more than one SREBP homolog in ascomycetes. However, in some fungi, not all SREBP homologs have been shown to be involved in the regulation of lipid metabolism or in other functions of the SREBP pathway. Basidiomycetes *X. dendrorhous* and *C. neoformans* harbor only one Sre1 homolog, which is involved in lipid homeostasis, which in turn regulates other processes, for example, carotenogenesis in *X. dendrorhous*. The regulation of carotenoid biosynthesis in *X. dendrorhous* by the transcription factor Sre1 is thus a novel function of the SREBP pathway and is a regulatory bridge between the biosynthesis of sterols and carotenoids in this species. The recent data regarding the SREBP pathway in *X. dendrorhous* contributes to our understanding about the regulation of carotenogenesis in this yeast and to our knowledge of SREBP pathways in fungi.

## Data Availability

Not applicable.

## Abbreviations

SREBP: Sterol Regulatory Element-Binding Protein
MVA: Mevalonate
bHLH-LZ: Basic-helix-loop-helix leucine zipper
ER: Endoplasmic reticulum
SCAP: SREBP cleavage-activating protein
S1P: Site-1 protease
S2P: Site-2 protease
SREs: Sterol regulatory elements
Insig: Insulin-induced gene
Dsc: Defective for SREBP cleavage
Sre1N: N-terminal bHLH-LZ domain of Sre1
